# EEG education in Brazil: a national survey of adult neurology residents

**DOI:** 10.1590/0004-282X-ANP-2021-0150

**Published:** 2021-11-30

**Authors:** Elora Sampaio Lourenço, Dora Pedroso Kowacs, Jay Raman Gavvala, Pedro André Kowacs, Fábio Augusto NASCIMENTO

**Affiliations:** 1 Instituto de Neurologia de Curitiba, Department of Neurology , Curitiba, PR, Brazil. Instituto de Neurologia de Curitiba Department of Neurology Curitiba PR Brazil; 2 Baylor College of Medicine, Department of Neurology, Houston, TX, United States . Baylor College of Medicine Department of Neurology Houston TX United States; 3 Massachusetts General Hospital, Department of Neurology, Boston, MA, United States . Massachusetts General Hospital Department of Neurology Boston MA United States

**Keywords:** Electroencephalography, Neurology, Epilepsy, Internship and Residency, Education, Eletroencefalografia, Neurologia, Epilepsia, Internato e Residência, Educação

## Abstract

**Background:**

In light of the established challenges of resident EEG education worldwide, we sought to better understand the current state of neurology resident EEG education in Brazil.

**Objective:**

To define Brazilian EEG practices including in-residency requirements for EEG training and competency.

**Methods:**

We assessed the perspectives of adult residents (PGY1-3) on EEG education and their level of confidence interpreting EEG with a 24-question online survey.

**Results:**

We analyzed 102 responses from 52 Brazilian neurology residency programs distributed in 14 states. There were 18 PGY1s, 45 PGY2s, and 39 PGY3s. Ninety-six percent of participants reported that learning how to read EEG during residency was very or extremely important. The most commonly reported barriers to EEG education were insufficient EEG exposure (70%) and ineffective didactics (46%). Residents believed that standard EEG lectures were the most efficient EEG teaching method followed by interpreting EEG with attendings’ supervision. Roughly half of residents (45%) reported not being able to read EEG even with supervision, and approximately 70% of all participants did not feel confident writing an EEG report independently.

**Conclusion:**

Despite the well-established residency EEG education requirements recommended by the Brazilian Academy of Neurology (ABN), there seems to be a significant lack of comfort interpreting EEG among Brazilian adult neurology residents. We encourage Brazilian neurology residency leadership to re-evaluate the current EEG education system in order to ensure that residency programs are following EEG education requirements and to assess whether EEG benchmarks require modifications.

## RESUMO

**Antecedentes:**

Diante dos desafios da educação em EEG estabelecidos em todo o mundo, buscamos compreender melhor o estado atual da educação em EEG durante a residência de neurologia no Brasil**.**

**Objetivo:**

Investigar práticas de EEG no Brasil, incluindo requisitos para treinamento e competência durante a residência de neurologia.

**Métodos:**

Avaliamos as perspectivas dos residentes (R1-3) de neurologia (adulto) sobre educação em EEG e nível de confiança ao interpretá-lo através de questionário online de 24 perguntas.

**Resultados:**

Foram analisadas 102 respostas de 52 programas de residência distribuídos em 14 estados. Dezoito R1s, 45 R2s e 39 R3s responderam à pesquisa. Noventa e seis por cento dos participantes relataram que aprender a ler EEG durante a residência é muito ou extremamente importante. As barreiras mais relatadas para educação em EEG foram exposição insuficiente ao EEG (70%) e didática ineficaz (46%). Os participantes apontaram aulas como método de ensino mais eficaz, seguido pela interpretação do EEG supervisionada pelos chefes. Aproximadamente metade dos residentes (45%) relatou não ser capaz de ler EEG mesmo com supervisão e cerca de 70% não se sente confiante para escrever um laudo de EEG de forma independente.

**Conclusões:**

Apesar dos requisitos estabelecidos pela Academia Brasileira de Neurologia (ABN) sobre ensino de EEG durante a residência, há significativa falta de confiança na sua interpretação pelos residentes de neurologia (adulto). Incentivamos as lideranças a reavaliar o sistema de educação para garantir que os programas de residência sigam requisitos de educação em EEG e se os benchmarks de EEG requerem modificações.

## INTRODUCTION

Brazilian adult neurology residency training consists of three years, the first of which focuses on internal medicine rotations. Recommendations posted by the National Medical Residency Commission in conjunction with the Brazilian Academy of Neurology (ABN) mandate that graduating adult neurology residents should be able to “know the indications of EEG and interpret it in the various neurological diseases”^1^. These expectations are similar in the U.S. as American graduating adult residents are recommended to be able to “interpret common EEG abnormalities, recognize normal variants, and create a report”^2^ . 

American data, however, suggests that a large portion of graduating neurology residents do not feel confident interpreting EEGs independently^3^^-^^5^. This issue is at least partially explained by a lack of consistency in teaching and evaluating residents during in-residency EEG training^6^. Nuances of EEG teaching and learning in Brazil have not yet been explored. Herein, we sought to define current neurology and EEG practices in Brazil including in-residency requirements for EEG training and competency. 

## METHODS

We assessed adult neurology residents’ perspectives on EEG education and their level of confidence interpreting EEG with a 24-question online survey (Supplementary File 1) hosted on Survey Monkey. The survey was adapted from a recently published North-American study^5^ and consisted of five questions focused on participants’ demographics, nine questions focused on perspectives on EEG education, and ten questions focused on level of confidence interpreting EEG, writing an EEG report, and identifying select EEG findings. All questions were close-ended except for one, which asked for suggestions to improve EEG teaching. 

The survey was disseminated among adult neurology residents (PGY1-3) through email and social media. There are currently 89 adult neurology residency programs in Brazil located in 20 of the 26 Brazilian states and one Federal District^7^. Trainees undergoing specialization in neurology, rather than neurology residency, were excluded. Invitations to participate in this study were sent by email to (i) residency program directors, whose contact information was obtained from the Medical Residency Commission, and (ii) directly to residents through partnership with the ABN. Data collection was performed from November 2020 to January 2021. This study was approved by the Neurological Institute of Curitiba (INC) institutional regulatory board (IRB).

## RESULTS

**Figure 1.**
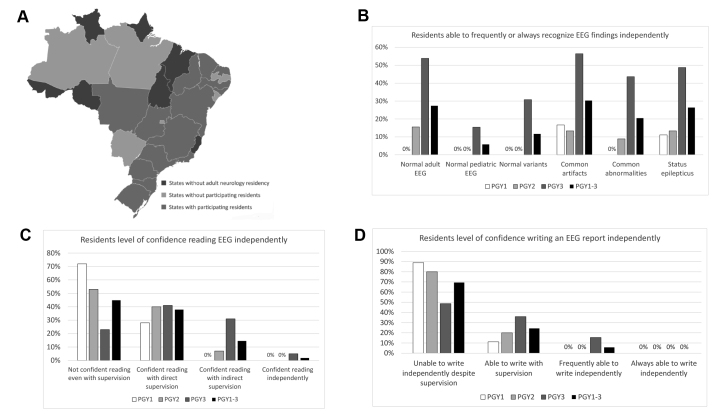
Summary of survey findings.

We obtained responses from 123 subjects, 21 of whom were excluded for not being residents. We analyzed the remaining 102 responses. Participating residents were from 52 different Brazilian adult neurology residency programs distributed in 14 states (Figure 1A). Among all participants, there were 18 PGY1s, 45 PGY2s, and 39 PGY3s. Main results are summarized in Figure 1. We classified hospitals affiliated to residency programs according to ^8^^,^^9^ a) level of care: secondary (specialized care) or tertiary (highly specialized care); b) ownership status: public, private, or charity; and c) size: special (over 500 beds), large (151 to 500 beds), medium (51 to 150 beds), and small (less than 51 beds). This data is summarized in (Supplementary File 2. 

### Residents’ perspectives on EEG education

The vast majority of residents (96%) reported that learning how to read EEGs during residency was very important or extremely important. Similarly, virtually all residents (98%) disagreed that learning to read EEG during residency is important only if one is pursuing further training in neurophysiology and/or epilepsy. 

Residents considered insufficient EEG exposure (70%), ineffective didactics (46%), suboptimal supervision from residents/fellows (34%), insufficient responsibility to read EEG during rotations (34%), and inability to link EEG learning to direct patient care (29%) as the most prominent barriers to learning EEG. Through qualitative assessment, participants also mentioned “not having access to EEG tracings”. Intuitively, the most commonly reported solutions to improvement in EEG education comprised increased EEG exposure (78%) and more efficient didactics (58%). Residents highlighted the importance of “increased supervision from the ABN in order to ensure that EEG is being taught” and one participant emphasized the challenge arising from “EEG learning not being required”. Many residents asked for “more EEG learning material” with “video, books, and online courses”. Participants were asked to rate different EEG teaching methods by level of efficiency. In a descending order, these were the methods reported: standard EEG lectures, interpreting EEG with attendings’ supervision, interpreting EEG with fellows’ supervision, interpreting independently followed by attendings’ review, and interpreting independently followed by fellows’ supervision.

In terms of ensuring competency in reading EEG, participants considered the number of EEGs read (85%) and number of hours reading EEG (56%) as the best objective measures. Alternative methods reported comprised written (37%) and oral (24%) EEG tests. Further, most residents (75%) reported that reading more than 50 EEGs is required to ensure competency in EEG.

### Residents’ level of confidence in EEG

Participants were asked whether they felt confident identifying particular EEG findings and/or states. Residents stated not being able to independently recognize a normal awake and sleep EEG in adults (35%) and children (72%), status epilepticus (26%), common artifacts (31%), common abnormalities (34%), and common normal variants (46%). Additional data regarding residents’ level of confidence in EEG interpretation is summarized in Figure 1B.

Overall, almost half of residents (45%) reported not being able to read EEG even with supervision. Roughly 70% of residents did not feel confident writing an EEG independently. This data is summarized in Figure 1 C-D. Lastly, 91% of residents felt null to little confidence explaining an EEG and its findings to patients and/or students. This percentage also varied between PGY classes: 94% (PGY1), 96% (PGY2), 85% (PGY3).

## DISCUSSION

This is the first nationwide study assessing adult neurology resident EEG education in Brazil. Responses were received from approximately 10% of all Brazilian adult neurology residents based on latest available demographic data^10^. Virtually all participants (96%) felt as though learning to read EEG in residency was important. This seems to be in line with resident perception data highlighted in other studies^3^^,^^5^. 

Despite the overwhelming evidence suggesting that EEG education is perceived as highly important by residents, trainees continue to report not being able to read or write EEG reports even with supervision. In our study, roughly half of residents (45%) reported not being able to read EEG even with supervision. This figure progressively decreased from PGY1 to PGY3; nonetheless, it remained high even among PGY3s (23%). In terms of writing EEG reports, almost 70% of all participants felt unable to do so independently. Although this percentage progressively declined from PGY1 to PGY3, it was still high in the PGY3 subgroup (49%). Overall, a significant portion of residents did not feel confident recognizing multiple EEG findings even with supervision.

A smaller, single-center cohort of American adult neurology residents showed similarly alarming results: 43% reported not being able to read EEG even with supervision, and 21% not being able to write an EEG report independently^5^. These residents were not categorized by postgraduate year level. An additional American survey-based study including 15 adult neurology PGY4s assessed these residents’ level of confidence in “interpreting common EEG abnormalities and creating a report” - the median was 67%^4^. Lastly, according to the last triannual American Academy of Neurology (AAN) survey, only 37.3% of graduating adult neurology residents felt confident performing or interpreting EEG independently^3^.

We also asked for participants’ opinions on multiple aspects of EEG education. The most commonly reported barriers to EEG learning were insufficient EEG exposure (70%) and ineffective didactics (46%). The former seems to be an issue in the American residency EEG education system as well^5^. Our study results also showed that participants believed that the two most efficient ways to teach EEG are standard EEG lectures and interpreting EEG with attendings’ supervision. Participants considered the number of EEGs read (85%) and number of hours reading EEG (56%) as the best objective measures to ensure EEG reading competency. Three-quarters of residents agreed that reading more than 50 EEGs would be required to ensure competency. A combination of didactic lectures and reading EEG with supervision were also considered the most efficient ways to teach EEG by an American cohort of adult neurology residents^5^. The latter cohort also felt as though the number of EEGs reviewed and number of hours spent reviewing EEG were the most effective competency measures^5^. 

Our study has limitations. We surveyed approximately 10% of all adult neurology residents in Brazil, a small number relative to the entire adult neurology trainee cohort in the country. Additionally, we only sampled residents from 14 out of the 20 Brazilian states where there are adult neurology residency program(s). Further, our survey might have suffered selection bias where residents who felt as though they had received inadequate EEG training were more likely to participate in our study. As a result, one needs to be cautious when extrapolating our study data to all residents on a national level.

In Brazil, graduating adult neurology residents are expected to “know the indications of EEG and interpret it in the various neurological diseases”^1^. Moreover, residents must read 250 EEGs throughout their training (50 as PGY2 and 200 as PGY3) and earn a minimum of 320 credit hours in clinical neurophysiology (EEG and EMG) to be eligible to graduate^1^. The importance of ensuring that graduating residents are able to read EEG independently arises from the fact that, in Brazil, EEGs may be read by physicians with or without training in clinical neurophysiology or epilepsy^11^ - presumably most frequently by general neurologists. Scenarios where EEGs are read by physicians who are not entirely comfortable with interpreting EEGs share intrinsic concerns especially in the realm of patient care^12^. 

In spite of the established requirements for residency EEG education^1^, there seems to be a significant lack of comfort interpreting EEG among Brazilian adult neurology residents. Based on our survey data, the two most commonly reported barriers to learning EEG were insufficient exposure and ineffective didactics. We hypothesize that these issues may arise from clinically demanding residency training programs as well as a lack of consistency in teaching and evaluating residents among the programs. Future studies including a larger sample of residents would be warranted to potentially confirm our findings and further analyze underlying issues precluding optimal resident EEG education.

We encourage Brazilian residency leadership to re-evaluate the current EEG education system in order to (i) ensure that residency programs are following ABN requirements^1^ and (ii) assess whether EEG benchmarks require modifications. Possible improvements may include increasing resident EEG exposure, optimizing didactics, and maximizing faculty time reviewing studies with trainees. It would also be reasonable - especially if our findings are confirmed by more comprehensive studies - to consider enhancing specific resident EEG milestones and creating objective and standardized methods of competency evaluation. We believe that improving resident EEG education results in optimization of nationwide EEG practices thereby ultimately improving patient care.

## SUPPLEMENTARY FILES

Supplementary File 1. Survey utilized in this study.

Supplementary File 2. Classification of hospitals affiliated with participating residency programs based on location, level of care, ownership status, and size.
