# Supplementary material for: EEG education in Brazil: a national survey of adult neurology residents
Source: Arq Neuropsiquiatr. 2021 Nov 30;80(1):43–7. doi: 10.1590/0004-282X-ANP-2021-0150 (PMC9651503; doi:10.1590/0004-282X-ANP-2021-0150)
Supplement: Supplementary File 1. [file 1678-4227-anp-80-01-043-s1.pdf]

https://doi.org/10.1590/0004-282X-ANP-2021-0150

EEG perspectives

Personal information

1. Please enter the initials of your full name. This information is only necessary to prevent the person from answering this questionnaire more than once.
2. Please enter a contact email.
3. Please enter a contact number (mobile).
4. Please enter the name of your institution.
5. What is your training level?

- PGY1
- PGY2
- PGY3
- Other (please specify)

EEG perspectives

EEG Education

6. How would you describe learning to read EEGs during neurology residency?

- Extremely important
- Very important
- Somewhat important
- Not so important
- Not at all important

7. Learning to read EEGs during neurology residency becomes important only if one is pursuing a neurophysiology/epilepsy fellowship.

- Agree
- Disagre

8. How confident are you that you can accurately read an EEG independently?

- Cannot read even with supervision
- Can read with direct supervision
- Can read with indirect supervision
- Can read independently without supervision

9. What is your confidence level in explaining an EEG procedure and results to a patient/caregiver/student?

- Extremely confident
- Very confident
- Somewhat confident
- Not so confident
- Not at all confident

10. What is your confidence level in using appropriate terminology related to EEG (e.g., montage, amplitude, frequency)?

- Extremely confident
- Very confident
- Somewhat confident
- Not so confident
- Not at all confident

11. Are you able to recognize normal EEG features of wake and sleep states in an adult patient?

- I always recognize these features independently without help from a supervisor
- I often recognize these features independently
- I recognize these features but need help from a supervisor
- I cannot recognize these features consistently

12. Are you able to recognize EEG patterns of status epilepticus?

- I always recognize these features independently without help from a supervisor
- I often recognize these features independently
- I recognize these features but need help from a supervisor
- I cannot recognize these features consistently

13. Are you able to recognize common EEG artifacts?

- I always recognize these features independently without help from a supervisor
- I often recognize these features independently
- I recognize these features but need help from a supervisor
- I cannot recognize these features consistently

14. Are you able to recognize common EEG abnormalities?

- I always recognize these features independently without help from a supervisor
- I often recognize these features independently
- I recognize these features but need help from a supervisor
- I cannot recognize these features consistently

15. Are you able to recognize normal EEG variants?

- I always recognize these features independently without help from a supervisor
- I often recognize these features independently
- I recognize these features but need help from a supervisor
- I cannot recognize these features consistently

16. Are you able to recognize normal EEG features of wake and sleep stages in children?

- I always recognize these features independently without help from a supervisor
- I often recognize these features independently
- I recognize these features but need help from a supervisor
- I cannot recognize these features consistently

17. Can you independently write an EEG report?

- I always independently generate a report without help from a supervisor
- I am often able to generate a report independently
- I need help from a supervisor to generate a report
- I cannot generate a report consistently

18. What do you consider barriers to learning EEG? Check all that apply.

- Insufficient exposure to EEG
- Inefficient EEG didactic lectures
- Subotimal education/supervision from attending and/or fellow
- Not a priority since I am not pursuing an EEG/epilepsy career
- Inability to link EEG learning to direct patient care (ie reading EEGs and caring for epilepsy patients at different times/rotations)
- Insufficient responsibility to read EEGs and formulate reposts during EEG rotation
- Other (please specify)

19. What would be the solutions to the barrier(s) selected in question 18?

- Increased exposure to EEG
- More efficient teaching methods
- Optimal education / supervision of residents or specialization / fellows
- Link EEG learning to direct patient care (for example, EEG interpretation and care of patients with epilepsy at different times/stages)
- Responsibility to actually read EEG and report EEG forms during internship
- Other (specify)

20. What do you think would be the most efficient way(s) to teach EEG? Please rank the following

- Standard EEG lectures
- Reading EEGs with concomitant supervision from attending
- Reading EEGs with concomitant supervision from fellow
- Reading independently followed by review with attending
- Reading independently followed by review with fellow

21. In your opinion, what would be the most effective measure to ensure competency in reading EEGs?

- Number of EEGs reviewed
- Number of hours spent reviewing EEGs
- EEG written test
- EEGOral examination
- Other (pleasespecify)

22. What would be the mininum number of EEGs reviewed to become competent in reading EEGs?

- 1-10
- 11-20
- 21-30
- 31-40
- 41-50
- > 50

23. What would be the minimum number of hours spent reviewing EEGs to become competent in reading EEGs?

- 1-40
- 41-80
- 81-120
- 121-160
- 161-200
- 201-240
- 241-280
- 281-320
- > 320

24. Please provide us your suggestions on how to improve EEG teaching.
