# Supplementary material for: EEG education in Brazil: a national survey of adult neurology residents
Source: Arq Neuropsiquiatr. 2021 Nov 30;80(1):43–7. doi: 10.1590/0004-282X-ANP-2021-0150 (PMC9651503; doi:10.1590/0004-282X-ANP-2021-0150)
Supplement: Supplementary File 2. [file 1678-4227-anp-80-01-043-s2.pdf]

https://doi.org/10.1590/0004-282X-ANP-2021-0150

Supplementary File 2. Classification of hospitals affiliated with participating residency programs based on location, level of care, ownership status, and size.

Location	Level of care	Ownership status	Size
Alagoas	Tertiary	Public	Large
Bahia	Tertiary	Private and charity	Large
Bahia	Tertiary	Private and charity	Large
Ceará	Tertiary	Public	Large
Ceará	Tertiary	Public	Large
Goiás	Tertiary	Public	Large
Goiás	Tertiary	Public	Large
Goiás	Tertiary	Private and charity	Large
Mato Grosso	Tertiary	Public	Large
Minas Gerais	Tertiary	Public	Large
Minas Gerais	Tertiary	Public	Large
Minas Gerais	Tertiary	Public	Large
Minas Gerais	Tertiary	Private and charity	Large
Paraná	Tertiary	Public	Large
Paraná	Tertiary	Private	Large
Paraná	Tertiary	Private and charity	Large
Paraná	Tertiary	Private	Medium
Paraná	Tertiary	Private and charity	Large
Paraná	Tertiary	Private and charity	Large
Paraná	Tertiary	Public	Large
Paraná	Tertiary	Private and charity	Large
Paraná	Tertiary	Private and charity	Large
Paraná	Tertiary	Public	Large
Pernambuco	Tertiary	Public	Large
Pernambuco	Tertiary	Public	Large
Pernambuco	Tertiary	Public	Large
Piauí	Tertiary	Public	Large
Rio de Janeiro	Tertiary	Public	Large
Rio de Janeiro	Tertiary	Public	Large
Rio Grande do Norte	Tertiary	Public	Large
Rio Grande do Sul	Tertiary	Charity	Large
Rio Grande do Sul	Tertiary	Private	Large
Rio Grande do Sul	Tertiary	Public	Large
Rio Grande do Sul	Tertiary	Private and charity	Large
Rio Grande do Sul	Tertiary	Public	Large
Rio Grande do Sul	Tertiary	Public	Large
Santa Catarina	Tertiary	Private and charity	Large
São Paulo	Tertiary	Public	Large
São Paulo	Tertiary	Private and charity	Large
São Paulo	Tertiary	Public	Large
São Paulo	Tertiary	Public	Large
São Paulo	Tertiary	Public	Large

Supplementary File 2. Cont.

Location	Level of care	Ownership status	Size
São Paulo	Tertiary	Public	Large
São Paulo	Tertiary	Public	Large
São Paulo	Tertiary	Private and charity	Large
São Paulo	Tertiary	Private and charity	Large
São Paulo	Tertiary	Public	Large
São Paulo	Tertiary	Private and charity	Large
São Paulo	Tertiary	Public	Large
São Paulo	Tertiary	Private and charity	Large
São Paulo	Secondary	Private	Small
São Paulo	Tertiary	Private and charity	Large
